# Exciton–Phonon Coupling Induces a New Pathway for Ultrafast Intralayer-to-Interlayer Exciton Transition and Interlayer Charge Transfer in WS_2_–MoS_2_ Heterostructure: A First-Principles Study

**DOI:** 10.1021/acs.nanolett.4c01508

**Published:** 2024-06-18

**Authors:** Yang-hao Chan, Mit H. Naik, Jonah B. Haber, Jeffrey B. Neaton, Steven G. Louie, Diana Y. Qiu, Felipe H. da Jornada

**Affiliations:** † Institute of Atomic and Molecular Sciences, Academia Sinica, Taipei 10617, Taiwan; ‡ Physic Division, National Center of Theoretical Sciences, Taipei 10617, Taiwan; § Department of Physics, University of California, Berkeley, California 94720-7300, United States; ∥ Materials Sciences Division, Lawrence Berkeley National Laboratory, Berkeley, California 94720, United States; ⊥ Department of Physics, University of California, Berkeley, California 94720-7300, United States; # Department of Mechanical Engineering and Materials Science, Yale University, New Haven, Connecticut 06520, United States; 7 Department of Materials Science and Engineering, Stanford University, Stanford, California 94305, United States

**Keywords:** exciton−phonon coupling, ultrafast charge transfer, WS_2_/MoS_2_ heterobilayer, relaxation time

## Abstract

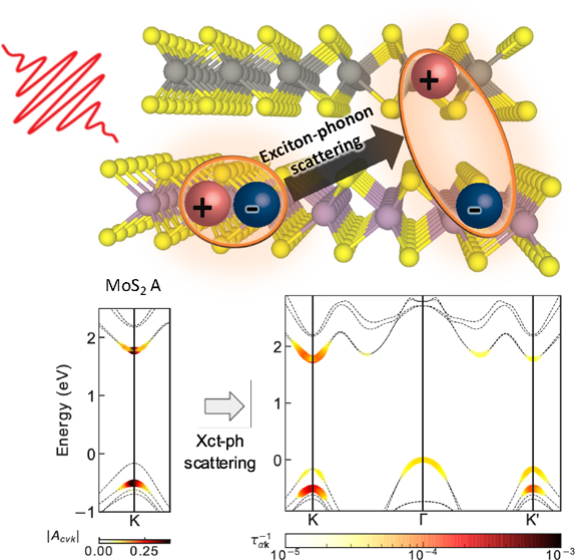

Despite the weak, van der Waals interlayer coupling, photoinduced charge transfer vertically across atomically thin interfaces can occur within surprisingly fast, sub-50 fs time scales. An early theoretical understanding of charge transfer is based on a noninteracting picture, neglecting excitonic effects that dominate optical properties of such materials. We employ an *ab initio* many-body perturbation theory approach, which explicitly accounts for the excitons and phonons in the heterostructure. Our large-scale first-principles calculations directly probe the role of exciton–phonon coupling in the charge dynamics of the WS_2_/MoS_2_ heterobilayer. We find that the exciton–phonon interaction induced relaxation time of photoexcited excitons at the *K* valley of MoS_2_ and WS_2_ is 67 and 15 fs at 300 K, respectively, which sets a lower bound to the intralayer-to-interlayer exciton transfer time and is consistent with experiment reports. We further show that electron–hole correlations facilitate novel transfer pathways that are otherwise inaccessible to noninteracting electrons and holes.

The freedom to stack quasi-two-dimensional (quasi-2D) van der Waals (vdW) materials introduces a vast parameter space for designing and engineering device properties, as well as exploring new physics by tuning electron correlations and order parameters through proximity effects.^1^ Stacked layers of transition metal dichalcogenides (TMDs) have attracted a lot of interest owing to their unique interplay of spin, valley, and optical chirality.^2^ Additionally, TMD heterostructures form semiconductors with type II band alignment. Optically excited intralayer excitons can scatter to lower-energy interlayer excitons with longer recombination times. The time scale and microscopic mechanism behind such exciton transfer processes are of fundamental interest and crucial for applications ranging from energy harvesting to quantum information.^3^

Recent pump–probe experiments have suggested that for the WS_2_/MoS_2_ heterobilayer such interlayer charge transfer can take place on a time scale of less than 50 fs.^4^ This exceptionally short charge transfer time is surprising, since it is well-understood that the valence band maximum (VBM) and conduction band minimum (CBM) from individual layers, at the *K* point, are only weakly hybridized. Phonon modes of the heterostructure also display negligible coupling, apart from very long wavelength acoustic modes. Furthermore, there is a momentum mismatch between the intralayer and interlayer exciton that would prevent a direct Coulomb-mediated charge transfer between the lowest exciton states of the two kinds. Later experiments on this system show that this ultrafast transfer is stacking angle independent^5−8^ and has a weak dielectric-environment^9^ dependence. Despite intense experimental and theoretical efforts,^10,11^ it has so far remained difficult to disentangle the complex experimental observations in vdW heterostructures^12−16^ owing in part to the lack of quantitatively predictive *ab initio* theories that treat vibrational and electronic correlation effects on the same footing.

Earlier theoretical work on WS_2_/MoS_2_ heterostructures based on time-dependent density functional theory (TD-DFT) suggested that charge transfer is initiated by coherent charge oscillations and completed by electron–phonon interactions.^17^ Nonadiabatic molecular dynamics (NAMD) approaches provided evidence supporting the role of quantum coherence^18^ and electron–phonon interactions.^19−23^ Excitonic effects have also been incorporated in recent NAMD studies by combining the sampling of the atomic motion from Born–Oppenheimer molecular dynamics with electronic excited-state calculations. The different excited-state potential energy surfaces are approximated either within TD-DFT, using parametrized range-separated hybrid functionals,^24^ or within many-body perturbation theory by solving the Bethe–Salpeter equation (BSE).^25^ In a recent work, a new excitonic channel for intralayer-to-interlayer charge transfers was discovered with TD-aGW calculations.^26^ Overall, these calculations uncovered important aspects of the microscopic mechanism beyond independent-particle interlayer charge transfer and suggest that a two-step relaxation process occurs in the vdW heterostructure, with excitonic effects aiding in the process.^24^ However, while promising, the aforementioned methods typically sacrifice the description of electronic correlations to obtain the coupled description of electrons and phonons. For instance, NAMD calculations are often still restricted to small supercells, which makes the spectrum of excitonic states artificially sparse^27^ and may lead to qualitatively different exciton decay pathways.

In this Letter, we study exciton–phonon scattering in a WS_2_/MoS_2_ TMD bilayer heterostructure including both electron–hole and exciton–phonon^28−30^ interactions fully from first-principles within the framework of many-body perturbation theory. Our computed relaxation time of the two lowest-energy intralayer excitons (the A excitons in the two layers) shows that exciton–phonon couplings are capable of inducing ultrafast charge transfer. Moreover, in contrast to the two-step transfer pathway proposed in earlier NAMD studies, we find a direct charge transfer pathway enabled by electron–hole correlations and intravalley scattering, with scattering rates in agreement with the sub-50 fs bleaching of optical signatures seen in experiments.^4,6,8,13^

We focus on the energetically favorable H_*h*_^*M*^ stacking WS_2_/MoS_2_ heterostructure with a twist angle of 60°. Figure 1(a) shows the electronic band structure calculated with the G_0_W_0_ approach as implemented in the BerkeleyGW software package.^31,32^ The band structure in the *K* valley clearly exhibits a typical type II band alignment. The first (topmost) valence band at the *K* valley is of WS_2_ character, while the second valence band is of MoS_2_ character. The next two valence bands also follow the same order. The lowest two conduction bands at the *K* valley both have MoS_2_ character but opposite spin. The next two conduction bands are of WS_2_ character. The system is an indirect band gap semiconductor with the VBM at Γ (which is of hybridized characters of the two layers) and the CBM at *K*. The direct band gap is 1.93 eV at the *K* and *K*′ valleys, where the valence band is about 160 meV lower than that of the VBM. This energy landscape provides a relaxation path for holes generated in the *K* valley. Furthermore, the mix of Mo and W orbital character at the Γ and Λ_min_ valleys suggests an intermediate state that can mediate an intralayer to interlayer charge transfer pathway. In panel (b) we show the exciton dispersion by solving the BSE within the GW-BSE method^31,33^ for excitons with finite center-of-mass (COM) momentum **Q**.^34^ Due to the indirect band gap nature, the lowest-energy exciton has a finite COM momentum of **Q** = *K* and is of interlayer character. Our calculations show that the first intralayer bright exciton in the MoS_2_ layer has an energy of 1.97 eV, and the first intralayer WS_2_ bright exciton, which must have **Q** = 0, is located at 2.09 eV. We will refer to these bright excitons as the MoS_2_ and WS_2_ A exciton, respectively. The corresponding absorption peaks shown in Figure S2 in the Supporting Information (SI) agree well with the previous experiment,^4^ within 100 meV, and calculations.^35^ We note that both bright excitons lie above the quasiparticle continuum at 1.93 eV of the lower-energy interlayer excitons. Although bound interlayer excitons are abundant below the continuum, they only couple weakly to light. In the inset of Figure 1(a), we show the quasiparticle band structure overlaid with the spin expectation value. The spin-split bands at the *K* valley are a consequence of strong spin–orbit coupling. Due to the spin-polarization near the *K* valley, the bright MoS_2_ A exciton consists of electrons from the first conduction band and holes from the second valence band, counted from the Fermi energy downward, and the WS_2_ A exciton consists of electrons from the fourth conduction band and holes from the first valence band. We emphasize that the internal spin structure of each exciton is also relevant to selection rules for exciton–phonon couplings, which is crucial to understand exciton scattering pathways.^36^

**Figure 1:**
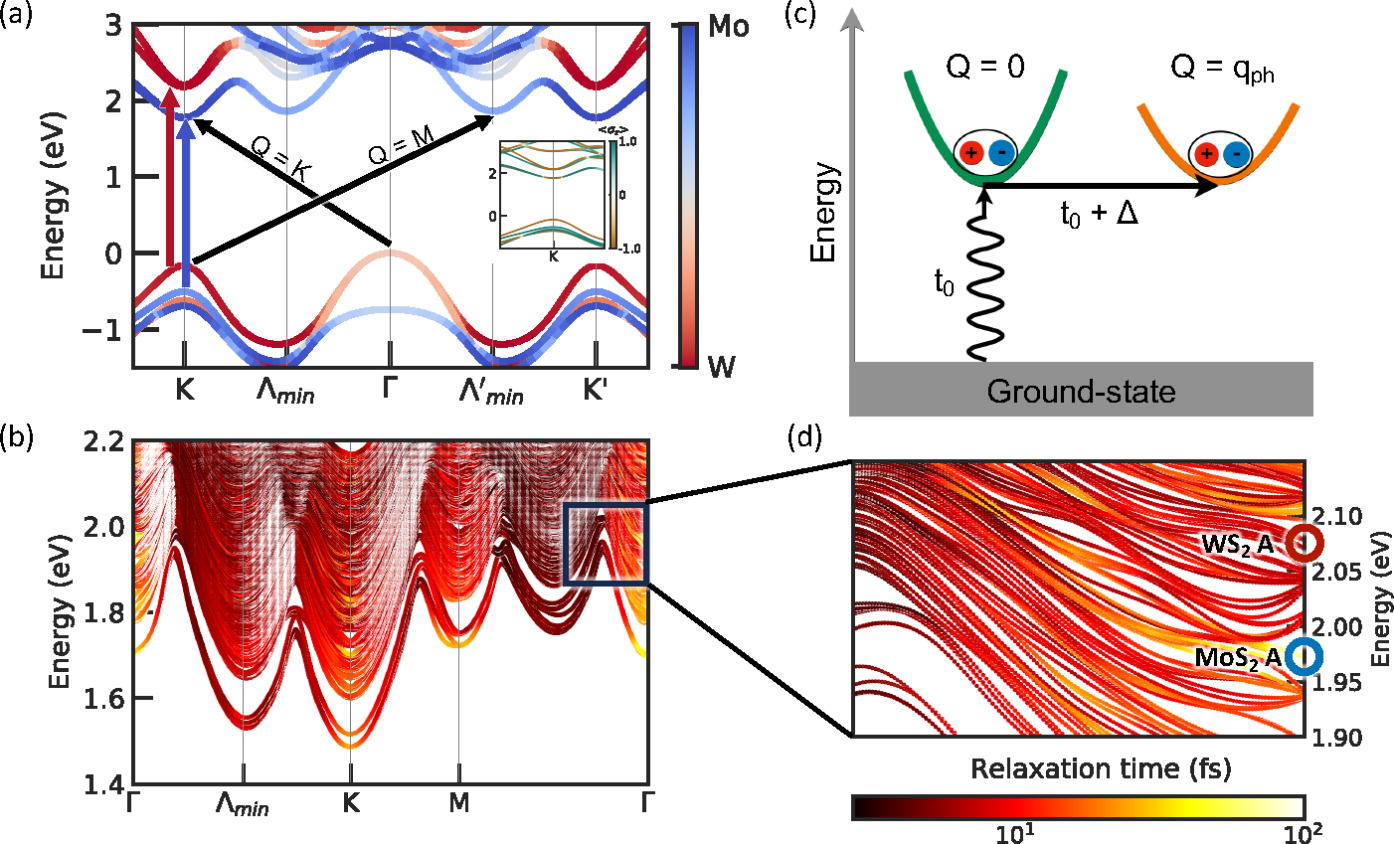
(a) Electron band dispersion of the WS_2_/MoS_2_ heterostructure overlaid with a color scale proportional to the contribution of Mo and W atoms to the projected component of the wave function squared. Interband transitions corresponding to excitons with center of mass momentum **Q** = *K* and **Q** = *M* are shown with the labeled arrows. The blue and red arrows indicate transitions corresponding to MoS_2_ and WS_2_ A excitons with **Q** = Γ, respectively. The inset shows a color map of the *z*-component of the spin expectation values of states near the *K* valley. (b) Exciton dispersion of both bound and resonant states of the WS_2_/MoS_2_ heterobilayer along a path of exciton center of mass momentum. Color indicates the value of the relaxation time due to exciton–phonon coupling at 300 K. (d) A zoom-in of panel (b) around the MoS_2_ and WS_2_ A excitons, which are indicated by blue and red circles, respectively. (c) Schematic representation of phonon-mediated exciton scattering process. A **Q** = Γ exciton forms at *t*_0_ and relaxes to an exciton with **Q** = **q**_ph_ at a later time *t*_0_ + Δ due to exciton–phonon interactions.

We study exciton kinetics in the conceptual framework of the Boltzmann equation, where exciton coherences are ignored and only exciton population dynamics is considered, including exciton–phonon interaction as the only scattering terms.^37,38^ Exciton scattering rates (inverse of the relaxation time) are evaluated from the imaginary part of exciton self-energy as introduced in previous work^28,29,36,39^ and in the SI. In Figure 1(c), we illustrate a typical scattering process that brings an exciton with zero COM momentum to a COM momentum of **q**. We emphasize this quantity gives the rate of an exciton being scattered from one state to all other states via the exciton–phonon coupling, and it is not between two specific states. In Figure 1(b), we show a color map of the exciton–phonon relaxation times in the heterostructure at 300 K. Our calculation shows that the MoS_2_ A exciton has a relaxation time of 67 fs, while the WS_2_ A exciton has a shorter relaxation time of 15 fs. If we take the computed relaxation time to be dominantly due to transitions to exciton states of interlayer character (see below), our results are consistent with the interpretation of the ultrafast charge transfer time observed in the experiments^4,6,8,13^ and with exciton–phonon interactions dictating the observed ultrafast optical response. The temperature dependence of the line width for both A excitons and the excitonic effects are shown in Figure S3.

Figure 1(d) shows a close-up of the region where the two A excitons are located. Since both excitons are in the continuum of the interlayer excitations, one would expect that the available phase space for exciton–phonon scattering is abundant and their relaxation times would be comparable. Yet, we find that the MoS_2_ A exciton has a relatively long lifetime compared to the WS_2_ A exciton. To understand this result, we analyze momentum- and state-resolved exciton–phonon couplings in Figure 2. The coupling matrix element *G*_*S*′*Sν*_(**Q**, **q**) encodes the probability amplitude for an exciton initially in state (*S*, **Q**) to scatter to state (*S*′, **Q** + **q**) through the emission or absorption of a phonon (ν, **q**). In Figure 2(a) and (b), we show color maps of the absolute value of band-resolved exciton–phonon coupling strength between the MoS_2_ A exciton and WS_2_ A exciton and other states summed over all phonon branches. We observe that both excitons are strongly coupled to **Q** = *M* excitons. Excitons with a COM momentum near **Q** = *K* also have appreciable coupling matrix elements. **Q** = *M* excitons consist of electrons at Λ_min_^′^ and holes at *K*, which we will denote as (*c*Λ_min_^′^, *vK*) pairs, while **Q** = *K* excitons can be either (*cK*′, *vK*) or (*cK*, *v*Γ) excitons.

**Figure 2:**
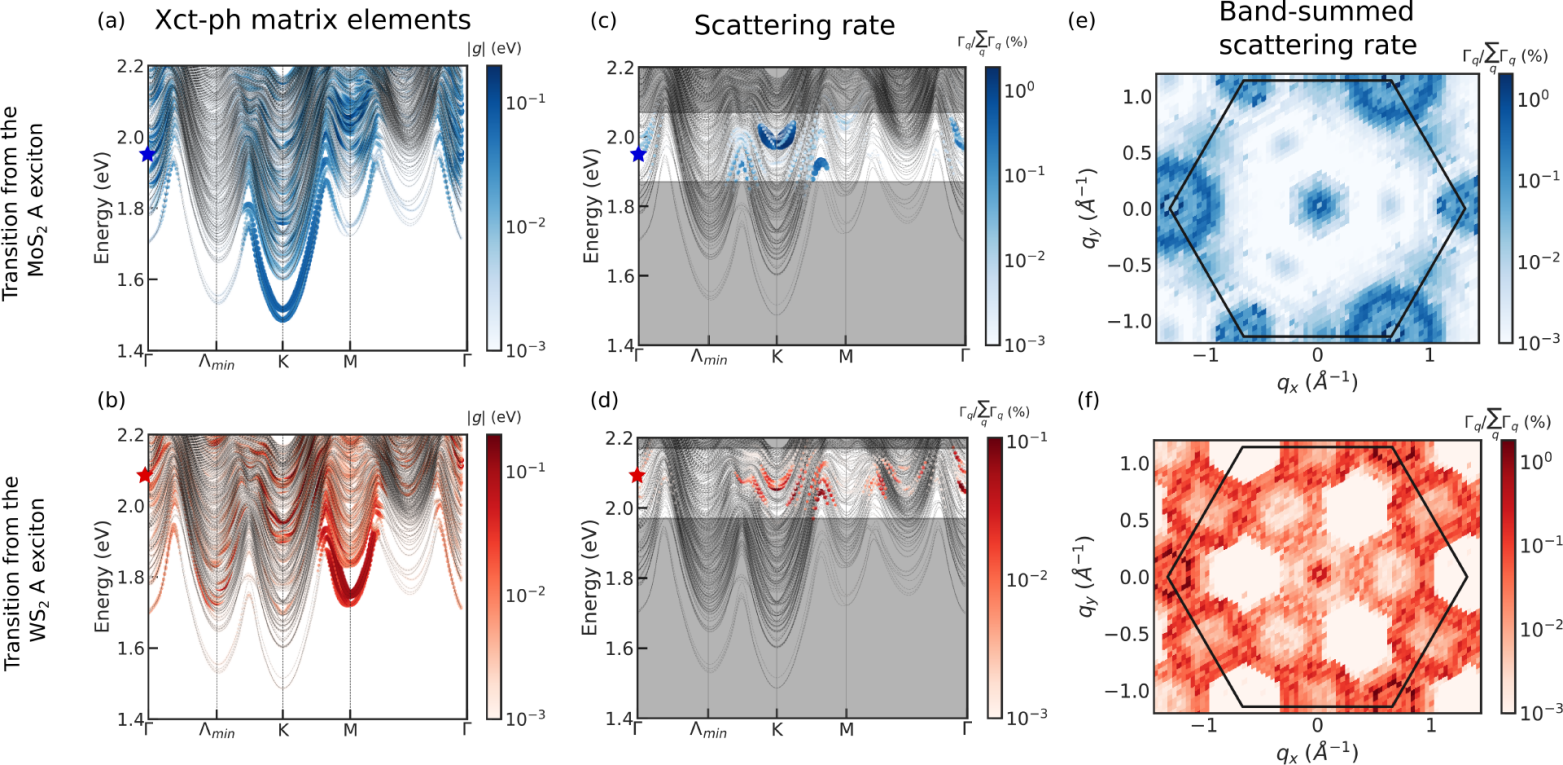
Exciton–phonon coupling strength (panels (a) and (b)) and phonon momentum **q**-resolved contribution to the total scattering rate for the MoS_2_ A exciton (blue star and panels (c) and (e)) and the WS_2_ A exciton (red star and panels (d) and (f)). (a) and (b) are the color maps of band-resolved amplitude of the exciton–phonon coupling matrix element along a high-symmetry path. The color scale and symbol sizes in (c) and (d) indicate the normalized contribution of the scattering rate from the starred state to other states summed over phonon modes. (e) and (f) show the normalized contribution to the scattering rate of the starred state from different phonon momenta **q**. Shaded region in (c) and (d) indicate the energy range where scatterings from A excitons are forbidden due to energy conservation.

The analysis based purely on exciton–phonon coupling matrix elements does not present a full picture of the relaxation time, which also includes energy conservation (imaginary part of eq S2). In Figure 2(c) and (d), we show exciton states resolved contributions to the scattering rate of the MoS_2_ (blue star) and WS_2_ (red star) A excitons along a high-symmetry path, respectively. The state-resolved scattering rate also appears in the Boltzmann equation and directly describes the microscopic kinetic process. Due to energy conservation conditions, only excitons in the energy window within one phonon frequency can contribute. Hence, for both layers, the A exciton relaxation time is dominated by scattering from **Q** = Γ to **Q** = *K* rather than **Q** = Γ to **Q** = *M*, which corresponds to the electron state of the exciton scattering between the *K* and *K*′ valleys.

We further analyze the scattering rate contributions resolved in the full Brillouin zone (BZ), as shown in Figure 2(e) and (f), which reveal important scattering channels for the MoS_2_ and WS_2_ A excitons, respectively, to any excitonic states via absorption and emission of phonons with wavevector **q**. These scattering rates in panels (e) and (f) correspond to the same initial states highlighted with stars in Figure 2 panels (c) and (d), respectively. We first studied the scattering pathway for an initially excited A exciton in MoS_2_. We observe that the pattern of contributions to the scattering rate shows a double-ring structure around the *K* valley. For the outer rings, our analysis shows that the final excitons have electrons and holes located mostly at the *K* and Γ points, respectively (see Figure S4(a) in the SI), indicating that the exciton–phonon scattering was mostly due to a change in the hole momenta. Because the valence states near Γ are layer-hybridized, we conclude that this scattering pathway is important for the ultrafast charge transfer observed in TMD heterobilayers.

On the other hand, scattering events associated with phonon wavevectors **q** in the inner ring around *K* are mostly associated with excitons wherein the electrons and holes are distributed at the *K* and *K*′ valleys, respectively (see Figure S4(b) in the SI). Importantly, we observe that the final holes change from their original character: mostly MoS_2_-like at the second valence band at *K* to mostly WS_2_-like at the VBM at *K*′. This channel indicates that the MoS_2_ A exciton can directly couple to interlayer excitons and cause Pauli blocking of the states associated with the WS_2_ A peak, leading to photobleaching of the WS_2_ absorption signal after optical pumping of the MoS_2_ A exciton.

A direct simulation of the photobleaching of the optical absorption spectrum in a pump–probe experimental setup can be done by solving for the time-dependent exciton populations from the exciton Boltzmann equation and taking a quasi-static approximation for the dielectric function.^38,40^ However, a full solution of the Boltzmann equation is computationally quite expensive due to the large amount of excitons and phonons involved. For instance, simply storing all the relevant exciton–phonon coupling matrix elements for our system on a uniform Monkhorst–Pack grid would amount to a file as large as hundreds of TB. As an approximation, we take the bleaching time as the scattering time from an A exciton to excitons with more than 1% weight on the first valence band with W characters at the *K* or *K*′ points. We find that the one-step scattering-event channel associated with the inner ring gives a bleaching time of the WS_2_ exciton of about 250 fs (Figure S5 in the SI). On the other hand, we also study a previously discussed two-step scattering mechanism, by which MoS_2_ A excitons are first scattered to excitons wherein the holes are distributed around to the VBM at Γ (the outer ring process), which subsequently scatters to interlayer excitons in an incoherent manner. We find that such a two-step process gives a bleaching time of the WS_2_ exciton of about 200 fs (Figure S6 in the SI). Similar scattering time was reported in ref (22) for the MoSe_2_/WSe_2_ bilayer. In ref (19) using nonadiabatic molecular dynamics simulations but neglecting excitonic effects, the authors conclude that holes placed in the second valence band at *K*, with initial MoS_2_ character, relax via both pathways detailed above on time scales of a few hundred femtoseconds. These individual time scales compare well, though are a bit longer than the bleaching time of the WS_2_ A exciton observed in experiment^4^ and, altogether, strengthen the case that multiple scattering mechanisms are important for ultrafast charge transfer in bilayer MoS_2_/WS_2_. We further give a detailed discussion of the temperature dependence of this effect in the SI.

Next, we focus on the dynamics of the WS_2_ A exciton. Figure 2(f) shows that the WS_2_ A exciton can scatter by emission or absorption of phonon wavevectors **q** over a larger region of the BZ. In particular, scatterings via phonons with **q** ∼ *K*, **q** ∼ *M*, **q** ∼ 0, and **q** ∼ Λ_min_^′^ are all viable. Overall, exciton–phonon coupling matrix elements of the MoS_2_ and WS_2_ A excitons are of the same order of magnitude; the larger scattering phase space of the WS_2_ A exciton results in its shorter relaxation time.

After showing that exciton–phonon scattering can be fast enough to explain ultrafast charge transfer observed in a WS_2_/MoS_2_ heterostructure, an important follow-up question is to microscopically understand how excitons relax after being excited by an optical field and what is the role of electron–hole correlation in that relaxation. The question can be addressed by solving the exciton Boltzmann equation, from which the transient absorption spectrum can further be simulated;^38,40^ however, a full numerical solution remains out of reach. Here, we instead develop an approach to qualitatively understand the population redistribution rate of each independent-particle orbital associated with exciton–phonon interactions and also estimate the photobleaching time of the WS_2_ A exciton (see the SI). We define the band- and **k**-resolved quasiparticle redistribution rate of a quasi-electron state *c***k** with an initially occupied A exciton as
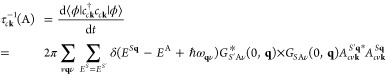
1where the expectation values are taken over an evolved state |ϕ⟩ starting from either the MoS_2_ or WS_2_ A exciton. *A*^*S***q**^ is the exciton envelope function of a state (*S*, **q**). Here, the scattered exciton COM momentum coincides with phonon momentum **q** since initial A excitons have zero COM momentum. A similar expression of the redistribution rate for valence electrons, along with its derivation, is given in the Supporting Information. In the case with nondegenerate exciton bands at specific **q**, eq 1 reduces to
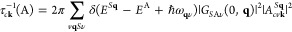
which can be understood as the exciton–phonon scattering rate weighted by the electron or hole components of the exciton envelope functions. In contrast to the independent-particle picture of electron–phonon scattering, we can see from the above expression that different scattering pathways become possible due to the nonlocal distribution of electron or hole amplitude in reciprocal space for excitonic states.

To see the effects of electron–hole interactions in the electron relaxation dynamics, we compare the computed quasiparticle redistribution rate with and without electron–hole interactions in Figure 3. We show the **k**- and band-resolved distribution of electrons and holes in the bilayer due to the presence of an initial photoexcited A exciton on MoS_2_ and WS_2_ in panels (a) and (e), respectively. We show the phonon-induced evolution in occupation of different quasiparticle states, including electron–hole interactions, by plotting τ_α**k**_^–1^(A) in panels (c) and (g), respectively. From Figure 3(c), it is clear that for an initial excitation of the MoS_2_ A exciton electrons mostly scatter within the same valley. Intervalley scattering to *K*′ and a remote Λ_min_^′^ occur with less probability. On the other hand, holes—initially at the second-highest valence band—scatter to both (i) the VBM at Γ and (ii) the two highest bands in the *K*′ valley. Since the Γ valley has a mixed character of both W and Mo atoms and the first band in the *K*′ valley is of W character, both scattering processes (i) and (ii) result in interlayer charge transfers.

**Figure 3:**
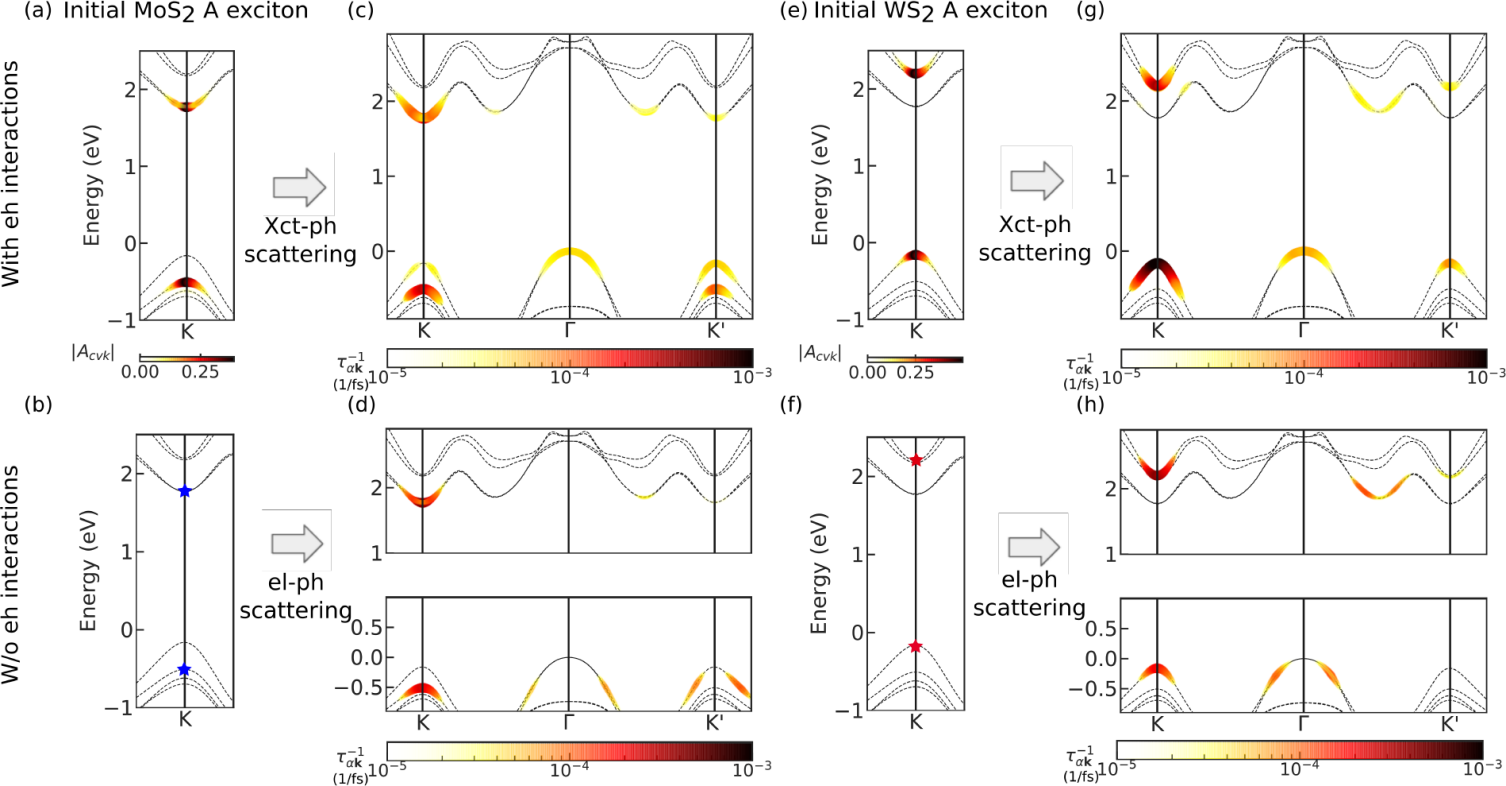
Evolution of the electrons and holes in bilayer MoS_2_/WS_2_ due to exciton–phonon interactions. Initial distribution of electrons and holes in the intralayer A exciton from MoS_2_ (a) and WS_2_ (e). Band- and *k*-resolved quasiparticle redistribution rate, τ_α**k**_^–1^ (A), due to exciton–phonon interactions, after the initial excitation of MoS_2_ (c) or WS_2_ (g). Blue (red) stars in panel (b) ((f)) represent the initial position of a free electron and hole at the *K* valley. (d) and (h) show the inverse of the redistribution time of independent electrons and holes due to electron–phonon scattering starting from the initial distribution in (b) and (f), respectively.

We also perform the corresponding calculations without excitonic effects (i.e., considering only electron–phonon interactions). If we start from the lowest-energy vertical transition (intralayer interband transitions) on MoS_2_ (blue stars in panel (b)), the corresponding quasiparticle scattering rate due to electron–phonon interactions is shown in panel (d). The scattering rate is much more limited in this case owing to the stricter energy-momentum conservation conditions in the noninteracting case. In contrast, excitons with energy close to the MoS_2_ A exciton can have a wide variety of energy and momentum distributions given their different possible internal structure, which allows for the coupling of a variety of states in the BZ. In particular, while holes transfer directly to the VBM at Γ and the *K*′ valley, it takes a secondary scattering event for this interlayer charge transfer if electron–hole interactions are not taken into account.

For the initially excited WS_2_ A exciton (Figure 3 (e)), the redistribution rate in Figure 3(g) indicates that scattered electrons have a wider distribution in the BZ than the MoS_2_ A exciton, which means that electrons are able to move across the whole BZ and is consistent with the analysis in Figure 2. For holes, on the other hand, we find that intravalley scattering is preferred. In the free electron–hole picture shown in Figure 3(h), we see that scattering of conduction electrons to a remote Λ_min_^′^ has a higher intensity, which is a consequence of the stronger electron–phonon coupling between the *K* and the Λ_min_^′^ valley. Valence electrons scatter mostly to the side of the Γ valley again due to energy conservation conditions. Comparing these two pictures, we can draw a conclusion that scattering in the exciton picture tends to redistribute charge over a wider range in the BZ, similar to that in the MoS_2_ A exciton case. We suggest this is a result of the correlated nature of excitons.

In conclusion, our first-principles calculations reveal the rich and ultrafast phonon-mediated exciton scattering channels in a prototypical TMD bilayer structure of WS_2_/MoS_2_. We show that the MoS_2_ A exciton has a relaxation time of about 67 fs and WS_2_ has a relaxation time of about 15 fs at 300 K. Moreover, we show that the ultrafast interlayer charge transfer takes place through a multiplicity of channels and that two-step scattering processes play a significant role: for an initially excited A exciton in MoS_2_, we predict such channels to cause a photobleaching of the absorption signal at the A exciton resonance in WS_2_ in about 200 fs. Our band BZ-resolved analysis further reveals that, upon excitation of the MoS_2_ A exciton, the relaxation primarily involves exciton scatterings which transfer the hole from the *K* point to the Γ region of the BZ, while, for the WS_2_ A exciton, it involves the scattering of the electron primarily to a valley around the Λ valley. We expect these findings to inform novel ways of stacking, electronic hybridization, and many-body effects and be synergistically employed to tune charge and energy dynamics in TMD heterostructures and that the formalism described here may be used in future studies involving exciton transport in real time.

## Supporting Information Available

Computational details of GW-BSE, electron–phonon, and exciton–phonon couplings, supplementary figures of phonon dispersion, absorption spectrum, exciton line width, exciton envelope functions, estimation of bleaching time, electron–phonon coupling matrix elements, and derivation of the exciton population change rate (PDF)
