# Supplementary material for: Exciton–Phonon Coupling Induces a New Pathway for Ultrafast Intralayer-to-Interlayer Exciton Transition and Interlayer Charge Transfer in WS2–MoS2 Heterostructure: A First-Principles Study
Source: Nano Lett. 2024 Jun 18;24(26):7972–8. doi: 10.1021/acs.nanolett.4c01508 (PMC11229060; doi:10.1021/acs.nanolett.4c01508)
Supplement: Supplementary file 1 — nl4c01508_si_001.pdf [file nl4c01508_si_001.pdf]

Supporting Information of Exciton-phonon coupling induces a new pathway for ultrafast intralayer-to-interlayer exciton transition and interlayer charge transfer in WS_2-MoS_2 heterostructure: a first-principles study

Yang-hao Chan,[1,2,*] Mit H. Naik,[3,4] Jonah B. Haber,[3,4] Jeffrey B. Neaton,[3,4] Steven G. Louie,[3,4] Diana Y. Qiu,[5,†] and Felipe H. da Jornada[6,‡]

[1]*Institute of Atomic and Molecular Sciences, Academia Sinica, Taipei 10617, Taiwan*
[2]*Physics Division, National Center of Theoretical Sciences, Taipei 10617, Taiwan*
[3]*Department of Physics, University of California, Berkeley, California, 94720-7300, United States*
[4]*Materials Sciences Division, Lawrence Berkeley National Laboratory, Berkeley, California, 94720, United States*
[5]*Department of Mechanical Engineering and Materials Science, Yale University, New Haven, Connecticut 06520, United States*
[6]*Department of Materials Science and Engineering, Stanford University, Stanford, California 94305, United States*

Computational details

1. GW-BSE and electron-phonon calculation

DFT calculations are performed with the Quantum Espresso[1] package. We use LDA pseudopotentials[2] from the PseudoDojo pseudopotentials database[3,4]. For the ground state calculation, we use a $\mathbf{k}$-mesh of $12\times12\times1$ and a plane wave energy cutoff of 50 Ry. A vacuum of 17 Å is chosen to prevent spurious interactions between periodic images. Spin-orbit coupling is included in the DFT and many-body theory calculations by treating each Kohn-Sham state within a fully relativistic, two-component spinor formalism. The GW-BSE calculation is done in the BerkeleyGW package[5–7]. A k-point grid of $6\times6\times1$ with a subsampling of 10 points in the mini-Brillouin zone (BZ)[8] and dielectric energy cutoff 35 Ry and 6000 bands are used in GW calculation. The frequency-dependence of the dielectric screening is computed using the Hybertsen-Louie generalized plasmon pole model[5]. The direct band gap in our DFT calculation is 1.32 eV and the GW corrected direct band gap is 1.93 eV. BSE with finite exciton COM $\mathbf{Q}$ are solved on a uniform 48×48 grid including 4 conduction and 4 valence bands.

Phonon calculations are performed with the density functional perturbation theory method (DFPT) implemented in the Quantum Espresso package. We solve phonon and perturbed self-consistent potential on a uniform 48×48 $\mathbf{q}$-grid. The computed phonon dispersion is shown in Fig. S1, which agrees reasonably well with previous reports. The small imaginary frequencies we obtained are commonly seen in calculations on 2D materials, and are not surprising since we have not explicitly enforced sum rules to fulfill the rotational invariance of the system[9]. Nevertheless, we set these imaginary frequencies to zero in our calculations. Electron-phonon coupling matrix elements are computed with EPW package[10,11].

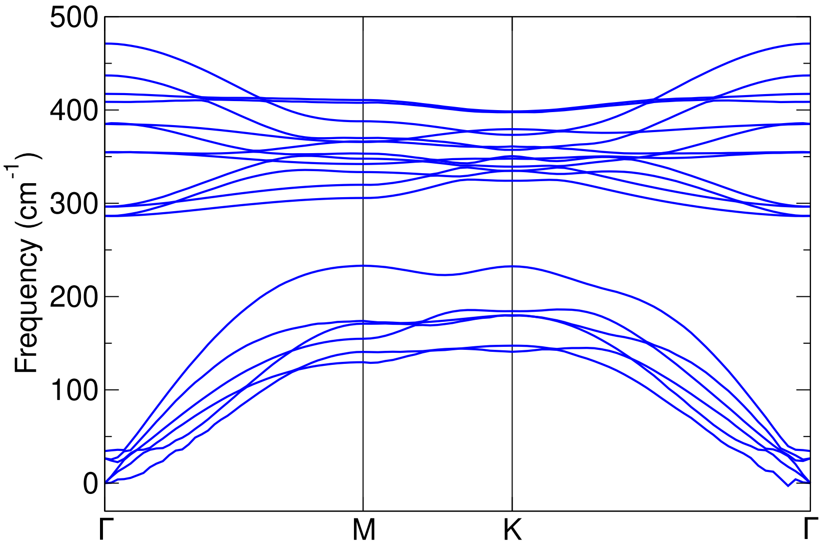

FIG. S1. Phonon dispersion of a WS_2/MoS_2 heterostructure.

2. Exciton-phonon self-energy calculations

Exciton-phonon coupling matrix elements can be written as a contraction of electron-phonon coupling matrix elements with the exciton envelope function[12–15], which are given as

$$G_{SS'\nu}(\mathbf{Q},\mathbf{q}) = \sum_{\mathbf{k}} \left[\sum_{vcc'} A^{S*}_{c\mathbf{k}+\mathbf{Q}+\mathbf{q},v\mathbf{k}} A^{S'}_{c'\mathbf{k}+\mathbf{Q},v\mathbf{k}} g_{cc'\nu}(\mathbf{k}+\mathbf{Q},\mathbf{q}) - \sum_{cvv'} A^{S*}_{c\mathbf{k}+\mathbf{Q},v\mathbf{k}-\mathbf{q}} A^{S'}_{c\mathbf{k}+\mathbf{Q},v'\mathbf{k}} g_{vv'\nu}(\mathbf{k}-\mathbf{q},\mathbf{q}) \right], \quad \text{(S1)}$$

where $A^{S}_{c\mathbf{k}+\mathbf{Q},v\mathbf{k}}$ is the envelope function of exciton S with a COM $\mathbf{Q}$ and $g_{c'c\nu}(\mathbf{k},\mathbf{q})$ is the electron-phonon coupling matrix elements which couples an electron of the band c and momentum $\mathbf{k}$ to the c' band via the phonon mode ν with momentum $\mathbf{q}$[13,14]. Physically, the first term in the bracket describes the scattering of conduction electrons in an exciton while the second term describes that of valence electrons.

To compute exciton-phonon coupling matrix elements it is important to ensure the gauge consistency between exciton envelope functions and electron-phonon coupling matrix elements. This is checked in our calculations by using the same DFT wavefunctions in the BSE kernel and the electron-phonon coupling matrix element calculations.

The lowest-order self-energy due to exciton-phonon couplings reads

$$\Sigma^{S\mathbf{Q}}(\omega) = \frac{1}{\mathcal{N}_{\mathbf{q}}} \sum_{S',\mathbf{q},\nu,\pm} \frac{|G_{S'S\nu}(\mathbf{Q},\mathbf{q})|^2 (N_{\nu\mathbf{q}} + \frac{1}{2} \pm \frac{1}{2})}{\omega - E^{S'\mathbf{Q}+\mathbf{q}} \mp \omega_{\nu\mathbf{q}} + i\eta}, \tag{S2}$$

where $E^{S'\mathbf{Q}+\mathbf{q}}$ is the exciton energy of a state with quantum number S' and a COM momentum $\mathbf{Q}+\mathbf{q}$; $\omega_{\nu\mathbf{q}}$ is the phonon frequency; $N_{\nu\mathbf{q}}$ is the Bose-Einstein occupation factor associated with the phonon, $\mathcal{N}_{\mathbf{q}}$ is the number of the wavevectors sampled in the Brillouin zone (BZ). Exciton scattering rates (inverse of the relaxation time) are evaluated from the imaginary part of exciton self-energy at $\omega = E^{S\mathbf{Q}}$ in Eq. S2. We compute the scattering rates with a tetrahedron method, which is equivalent to a calculation by performing a linear interpolation on both exciton energy and exciton-phonon coupling matrix elements.

3. Absorption spectrum

The computed absorption spectrum is given in Fig. S2. We find that the lowest absorption peak comes from an interlayer exciton. Its oscillator strength is however orders of magnitude smaller compared to MoS_2 or WS_2 intralayer excitons. The MoS_2 A exciton peak is at 1.97 eV while the WS_2 A exciton peak is at 2.09 eV. These results agree reasonably well with previous experiment[16] and calculations[17].

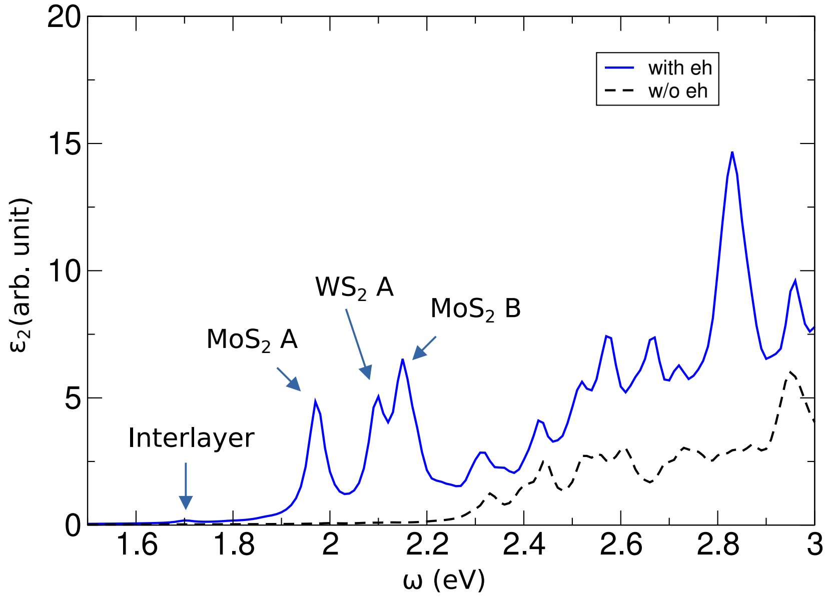

FIG. S2. Absorption spectrum of a WS_2/MoS_2 heterostructure. The blue line is the result with electron-hole interactions and the black dashed line is the result without electron-hole interactions. The lowest few prominent exciton peaks are labeled.

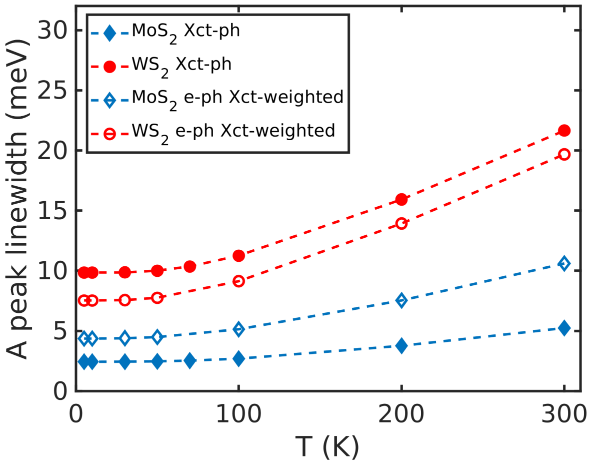

FIG. S3. Temperature dependent linewidth of MoS_2 (solid blue diamonds) and WS_2 (solid red dots) A excitons due to exciton-phonon couplings. Linewidth computed in the limit of zero exciton binding energy from the electron-phonon coupling weighted by exciton envelope functions are shown by the empty symbols.

4. Linewidth

The optical absorption linewidth encodes the information of exciton lifetimes and their coherence times. The temperature-dependent linewidth measured from experiments can help quantify, for instance, the exciton-phonon coupling strength. In the present theory framework, we approximate the linewidth as the imaginary part of Eq. S2, $\mathrm{Im}\Sigma^{S\mathbf{0}}(E_{S\mathbf{0}})$. This self-energy leads to a decay of the excited-state polarization; hence $\mathrm{Im}\Sigma^{S\mathbf{0}}(E_{S\mathbf{0}})$ directly contributes to T_2, the dephasing time[15]. The results for both MoS_2 and WS_2 A excitons are shown in Fig. S3. The exciton-phonon induced linewidth is about 5 meV and 22 meV at 300 K for MoS_2 and WS_2 A exciton, respectively. To explicitly see the effects of electron-hole interactions, we also compute the linewidth as a linear combination of electron-phonon (and hole-phonon) interaction weighted by the exciton envelope function, which we refer to as the exciton-weighted free electron-hole linewidth[18,19]. Although the exciton-phonon linewidth is within the same order of magnitude as that obtained from excited-weighted approach, there is no consistent relation between these two cases. For the MoS_2 A exciton, the simpler exciton-weighted electron-phonon linewidth shows a stronger temperature dependence and an overall larger linewidth, while the opposite is true for the for WS_2 A exciton. It is therefore necessary to compute exciton-phonon interactions directly for a correct description of exciton relaxations. Finally, we note that the linewidth of the MoS_2 A exciton increases to 5 meV in the MoS_2/WS_2 heterostructure compared to 2 meV in the monolayer at 300 K[15] while a 26 meV increase was reported in the experiment[20]. The underestimation might be owing to the fact that we do not consider defect scatterings and sample inhomogeneity in our calculations.

Envelope functions of a few key excitons

In Fig. S4, we show envelope functions of two excitons which contribute significantly to the MoS_2 A exciton scattering rate discussed in the main text. In particular, the envelope function with $\mathbf{Q} = (0.25, 0.25)$, which locates at the outer ring structure in Fig. 2 (e) in the main text is shown in Fig. S4 (a). Fig. S4 (b) shows the envelope function of one of the exciton locates at the inner ring with $\mathbf{Q} = (0.3125, 0.3125)$.

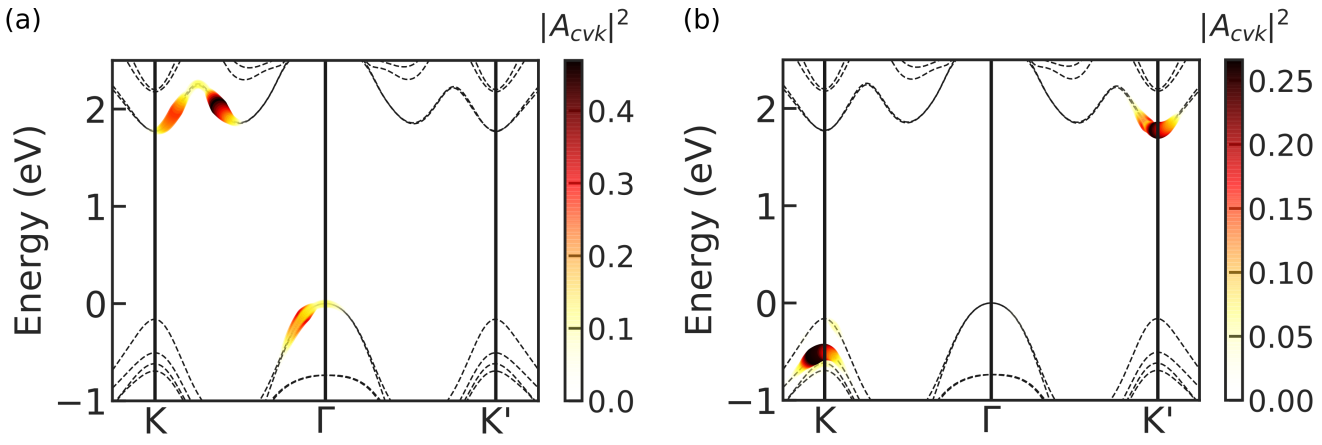

FIG. S4. Envelope functions along the high symmetry path of (a) one exciton in the outer ring structure in Fig. 2 (e) in the main text, which have holes located at Γ valley and of (b) one exciton in the inner ring structure.

Exciton Boltzmann equation, linewidth and transient absorption spectrum

Boltzmann equation for the exciton population can be derived from many-body perturbation theory[21] or from the Heisenberg equation with higher order correlations truncated and the ignorance of exciton coherences. Assuming a homogeneous real-space distribution without external forces, dynamics of exciton populations, $f_{n\mathbf{Q}}$, of an exciton state labeled by n and center-of-mass momentum (COM) $\mathbf{Q}$ are governed by,

$$\frac{\partial}{\partial t} f_{n\mathbf{Q}}(t) = \frac{2\pi}{\mathcal{N}_{\mathbf{q}}\hbar} \sum_{\mathbf{q},l,\nu} |G_{ln\nu}(\mathbf{Q},\mathbf{q})|^2 \Big\{ - f_{n\mathbf{Q}} \left(1 + f_{l\mathbf{Q}+\mathbf{q}}\right) \left[n_{\mathbf{q}\nu} D^{+}_{nl\mathbf{Q}\mathbf{q}} + \left(1 + n_{\mathbf{q}\nu}\right) D^{-}_{nl\mathbf{Q}\mathbf{q}} \right] + f_{l\mathbf{Q}+\mathbf{q}} \left(1 + f_{n\mathbf{Q}}\right) \left[(n_{\mathbf{q}\nu} + 1) D^{+}_{nl\mathbf{Q}\mathbf{q}} + n_{\mathbf{q}\nu} D^{-}_{nl\mathbf{Q}\mathbf{q}} \right] \Big\}, \tag{S3}$$

where the right-hand side is the collision operator which describes how band and momentum resolved exciton populations evolve in real-time due to microscopic exciton-phonon scattering events. In this expression, $n_{\mathbf{q}\nu}$ is the phonon occupation of a mode ν and crystal momentum $\mathbf{q}$, $\mathcal{N}_{\mathbf{q}}$ is the total number of $\mathbf{q}$ points and $G_{ln\nu}(\mathbf{Q},\mathbf{q})$ denotes the exciton-phonon coupling matrix element defined in the previous section. The energy conservation of scatterings is guaranteed through the delta function, $D^{\pm}_{nl\mathbf{Q}\mathbf{q}} = \delta(-\epsilon_{l\mathbf{Q}+\mathbf{q}} + \epsilon_{n\mathbf{Q}} \pm \hbar\omega_{\mathbf{q}\nu})$, where $\epsilon_{n\mathbf{Q}}$ is the exciton excitation energy and $\omega_{\mathbf{q}\nu}$ is the phonon energy. Each term in summation of the r.h.s of Eq. S3 describes a microscopic scattering process while the total scattering rate can be obtained from the sum of the first line.

In writing down the Boltzmann equation, exciton coherences are ignored. This assumption is often justified as the typical coherence decay time is much shorter than the population relaxation time or recombination time. On the other hand, the equation of motion of the full single exciton density matrix including exciton-exciton coherences can be derived from Heisenberg equation by keeping the coherences. To carefully check the effect of exciton coherences, we numerically solve both Boltzmann equation and a full density matrix equation for a monolayer MoS_2 from first-principles. We found that the effect of coherences on exciton dynamics can be safely ignored if we start from a single exciton state as the coherences generated from exciton-phonon scattering is small. Therefore, we only focus on the population transfers and the scattering rate described by the diagonal of the self-energy, Eq. 1 in the main text and assume incoherent dynamics in our analysis of photobleaching time.

Besides the linewidth discussed earlier, which is pertinent to experiments, we briefly discuss how we can simulate transient absorption spectrum in the current theory framework. The photobleaching time measured from the transient absorption spectrum can be obtained by directly simulating pump-probe experiments in a time-dependent approach, which however involves solving electron-hole coherences dynamics. Alternatively, transient absorption spectrum can be simulated from the time-dependent populations solved in Eq. S3 with the quasi-static assumption[22,23]. As solving Eq. S3 in WS_2/MoS_2 is numerically challenging due to large numbers of exciton momentum, bands, and phonon

modes involved, we turn the simple estimation discussed in the next section.

Estimation of bleaching time for different scattering path from MoS_2 A exciton

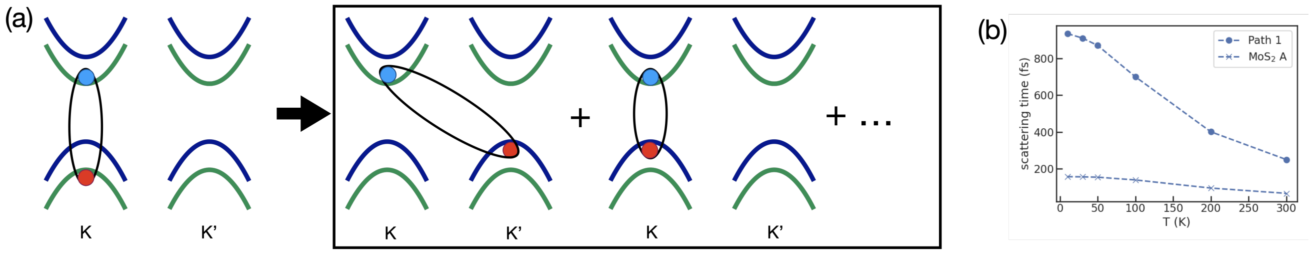

FIG. S5. (a) Scattering from MoS_2 A exciton to excitons with hole states on the first valence band at K or K', which is of WS_2 characters. (b) Total scattering time (blue crosses) and partial scattering time by summing over processes in (a) (blue dots) as a function of temperature.

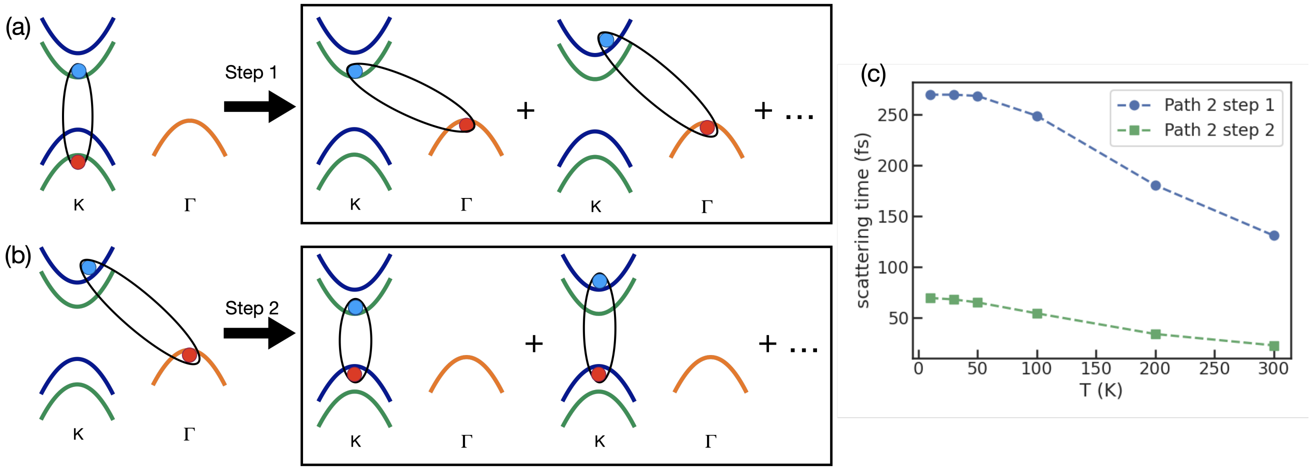

FIG. S6. A two-step scattering process of MoS_2 A exciton to excitons with hole states on the first valence at K or K'. (a) In step 1 MoS_2 A exciton is scattered to excitons with holes on topmost bands at Γ, which in step 2 are scattered to excitons with hole states on the first valence band at K or K'. (c) Scattering time of the two steps as a function of temperature for one of the intermediate exciton.

We provide an estimate of the bleaching time of the WS_2 A exciton by starting with a MoS_2 A exciton and consider scattering events to excitons in which the final excitons contain holes that overlap with those of the WS_2 A exciton. Multiple scattering pathways can cause the bleaching of WS_2 A exciton signal in the absorption spectrum, and we consider both one- and two-step processes.

We first consider a one-step bleaching process by summing over the scattering time from the initial MoS_2 A exciton to all other excitons in the BZ which have significant contributions on the highest valence band at K or K$'$. Specifically, we take into account excitons having more than 1% projection on those two bands within a 0.3 bohr^{-1} radius from K or K$'$. The temperature dependence of the scattering time is shown in Fig. S5, which gives an estimation of the bleaching time of a one-step scattering process.

Next, we consider a two-step process contribution to the bleaching time of WS_2 A exciton. While a full dynamics of excitons within, say, a Boltzmann dynamics-kind of approach would be desirable, it is not currently accessible given the large phase space we need to consider for this system, which requires about 200 bands and 2304 Q-points to label just the desired excitonic states. Therefore, consistently with a Boltzmann picture, though implemented in a

simplified fashion, we simulate the charge-transfer dynamics as a two-step process, neglecting memory effects between the intermediate states. More precisely, we neglect the phase information of the final excitons from the first scattering process and pick the most important state around Γ as the initial state for a second scattering event, also consistent with the picture of a Boltzmann-type process.

Specifically, we consider scattering time from MoS_2 A exciton to excitons with weights on VBM at Γ, and a subsequent scattering from one of the excitons involved in the first step to excitons with hole states on the highest valence band at K or K′. For the second step, the exciton shown in Fig. S4 (a) was taken as a representative state. The computed scattering time of the two steps is shown in Fig. S6 (c). We assume the two-step process does not involve memory effects, consistent with a Boltzmann picture, so the total scattering time is the sum of the two individual scattering times associated with the two sequential processes.

Derivation of the population change rate

In this section, we derive the population change rate $\frac{dP_\alpha}{dt}$, Eq. 2 in the main text. Starting from an initial state $|A,0\rangle$ with an A exciton and zero phonon occupation, we ask the expectation value of finding an electron or hole with a given quantum number at a later time t. We use time-dependent perturbation theory and treat excitons and phonons as independent particles among themselves and exciton-phonon coupling as a perturbation. The Hamiltonian is

$$\begin{aligned} H &= H_0 + H_{ex-ph} \\ H_0 &= \sum_{s\mathbf{Q}} E^{s\mathbf{Q}} a^\dagger_{s\mathbf{Q}} a_{s\mathbf{Q}} + \sum_{\mathbf{q}\nu} \hbar\omega_{\mathbf{q}\nu} b^\dagger_{\mathbf{q}\nu} b_{\mathbf{q}\nu} \\ H_{ex-ph} &= \sum_{ss'\nu\mathbf{Q},\mathbf{q}} G_{ss'\nu}(\mathbf{Q},\mathbf{q}) a^\dagger_{s\mathbf{Q}+\mathbf{q}} a_{s'\mathbf{Q}} \left(b^\dagger_{\mathbf{q}\nu} + b_{-\mathbf{q}\nu} \right), \end{aligned}$$

where $a_{s\mathbf{Q}}$ ($a^\dagger_{s\mathbf{Q}}$) is the annihilation (creation) operator of an exciton state s and COM $\mathbf{Q}$ and $b_{\mathbf{q}\nu}$ ($b^\dagger_{\mathbf{q}\nu}$) is the phonon annihilation (creation) operator of mode ν and momentum $\mathbf{q}$. $G_{ss'\nu}$ are the exciton-phonon coupling matrix elements defined in Eq. S1. To the first order of the perturbation the scattered state is $|\phi\rangle = e^{-iH_0t}U|A,0\rangle$, with

$$U = e^{iH_0t}e^{iHt} \backsimeq 1 - i\int dt_1 \hat{V}(t_1),$$

where we define the exciton-phonon coupling term in the interaction picture, $\hat{V} = e^{iH_0t}H_{ex-ph}e^{-iH_0t}$ and write the evolved state at time t,

$$\begin{aligned} i\int_0^t dt_1 \hat{V}(t_1)|A,0\rangle &= i\int_0^t dt_1 e^{iH_0t_1} H_{ex-ph} e^{-iH_0t_1}|A,0\rangle \\ &= i\int_0^t dt_1 e^{-iE^At_1} e^{iH_0t_1} \sum_{ss'\nu\mathbf{Q},\mathbf{q}} G_{ss'\nu}(\mathbf{Q},\mathbf{q}) a^\dagger_{s\mathbf{Q}+\mathbf{q}} a_{s'\mathbf{Q}} \left(b^\dagger_{\mathbf{q}\nu} + b_{-\mathbf{q}\nu} \right)|A,0\rangle \\ &= i\int_0^t dt_1 e^{-iE^At_1} e^{iH_0t_1} \sum_{s\mathbf{q}\nu} G_{sA\nu}(0,\mathbf{q})|s\mathbf{q},1_{\mathbf{q}\nu}\rangle \\ &= i\sum_{s\mathbf{q}\nu}\int_0^t dt_1 e^{i\left(E^{s\mathbf{q}}-E^A+\hbar\omega_{\mathbf{q}\nu}\right)t_1} G_{sA\nu}(0,\mathbf{q})|s\mathbf{q},1_{\mathbf{q}\nu}\rangle \\ &= i\sum_{s\mathbf{q}\nu} \frac{e^{i\left(E^{s\mathbf{q}}-E^A+\hbar\omega_{\mathbf{q}\nu}\right)t}-1}{i\left(E^{s\mathbf{q}}-E^A+\hbar\omega_{\mathbf{q}\nu}\right)} G_{sA\nu}(0,\mathbf{q})|s\mathbf{q},1_{\mathbf{q}\nu}\rangle. \end{aligned}$$

Together with the first term in the equation for U we get

$$\begin{aligned}
|\phi\rangle &= e^{-iE^A t}|A\rangle + e^{-iH_0 t}\sum_{s\mathbf{q}\nu}\frac{1-e^{i\left(E^{s\mathbf{q}}-E^A+\hbar\omega_{\mathbf{q}\nu}\right)t}}{(E^{s\mathbf{q}}-E^A+\hbar\omega_{\mathbf{q}\nu})}G_{sA\nu}(0,\mathbf{q})\,|s\mathbf{q},1_{\mathbf{q}\nu}\rangle \\
&= e^{-iE^A t}|A\rangle + e^{-iH_0 t}\sum_{s\mathbf{q}\nu}e^{i\left(E^{s\mathbf{q}}-E^A+\hbar\omega_{\mathbf{q}\nu}\right)t/2}\frac{e^{-i\left(E^{s\mathbf{q}}-E^A+\hbar\omega_{\mathbf{q}\nu}\right)t/2}-e^{i\left(E^{s\mathbf{q}}-E^A+\hbar\omega_{\mathbf{q}\nu}\right)t/2}}{(E^{s\mathbf{q}}-E^A+\hbar\omega_{\mathbf{q}\nu})}G_{sA\nu}(0,\mathbf{q})\,|s\mathbf{q},1_{\mathbf{q}\nu}\rangle \\
&= e^{-iE^A t}|A\rangle + e^{-iH_0 t}\sum_{s\mathbf{q}\nu}(-i)e^{i\left(E^{s\mathbf{q}}-E^A+\hbar\omega_{\mathbf{q}\nu}\right)t/2}\frac{\sin\left(E^{s\mathbf{q}}-E^A+\hbar\omega_{\mathbf{q}\nu}\right)t/2}{\left(E^{s\mathbf{q}}-E^A+\hbar\omega_{\mathbf{q}\nu}\right)/2}G_{sA\nu}(0,\mathbf{q})\,|s\mathbf{q},1_{\mathbf{q}\nu}\rangle \\
&= e^{-iE^A t}|A\rangle - i\sum_{s\mathbf{q}\nu}e^{-iE^A t}K(E^{s\mathbf{q}}-E^A+\hbar\omega_{\mathbf{q}\nu})G_{sA\nu}(0,\mathbf{q})\,|s\mathbf{q},1_{\mathbf{q}\nu}\rangle\,.
\end{aligned}$$

To ease the notation we define

$$K(E) = e^{-iEt/2}\frac{\sin Et/2}{E/2},$$

which has a long time limit given by[24]

$$\lim_{t\to\infty}|K(E)|^2 = \lim_{t\to\infty}2\pi t\delta(E).$$

The expectation value of finding a valence electron of quantum number $(h,\mathbf{k})$ is $\left\langle\phi\left|c^\dagger_{h\mathbf{k}}c_{h\mathbf{k}}\right|\phi\right\rangle$. We have

$$\begin{aligned}
c_{h\mathbf{k}}|\phi\rangle &= e^{-iE^A t}c_{h\mathbf{k}}|A\rangle - \sum_{s\mathbf{q}\nu}ie^{-iE^A t}K(E^{s\mathbf{q}}-E^A+\hbar\omega_{\mathbf{q}\nu})G_{sA\nu}(0,\mathbf{q})c_{h\mathbf{k}}|s\mathbf{q}\rangle|1_{\mathbf{q}\nu}\rangle \\
&= e^{-iE^A t}c_{h\mathbf{k}}\sum_{cv\mathbf{k}'}A_{cv\mathbf{k}'}c^\dagger_{c\mathbf{k}'}c_{v\mathbf{k}'}|0\rangle \\
&-\sum_{s\mathbf{q}\nu}ie^{-iE^A t}K(E^{s\mathbf{q}}-E^A+\hbar\omega_{\mathbf{q}\nu})G_{sA\nu}(0,\mathbf{q})c_{h\mathbf{k}}\sum_{cv\mathbf{k}'}A^s_{c\mathbf{k}'+\mathbf{q}v\mathbf{k}'}c^\dagger_{c\mathbf{k}'+\mathbf{q}}c_{v\mathbf{k}'}|0\rangle|1_{\mathbf{q}\nu}\rangle\,.
\end{aligned}$$

$$\begin{aligned}
\langle c_{h\mathbf{k}}\phi| &= \langle 0|e^{iE^A t}\sum_{cv\mathbf{k}'}A^*_{cv\mathbf{k}'}c^\dagger_{v\mathbf{k}'}c_{c\mathbf{k}'}c^\dagger_{h\mathbf{k}} \\
&+\sum_{s\mathbf{q}\nu}\langle 0|\langle 1_{\mathbf{q}\nu}|ie^{iE^A t}K(E^{s\mathbf{q}}-E^A+\hbar\omega_{\mathbf{q}\nu})G^*_{sA\nu}(0,\mathbf{q})\sum_{cv\mathbf{k}'}A^{s*}_{c\mathbf{k}'+\mathbf{q}v\mathbf{k}'}c^\dagger_{v\mathbf{k}'}c_{c\mathbf{k}'+\mathbf{q}}c^\dagger_{h\mathbf{k}}.
\end{aligned}$$

The expectation value is

$$\begin{aligned}
\left\langle\phi\left|c^\dagger_{h\mathbf{k}}c_{h\mathbf{k}}\right|\phi\right\rangle &= \sum_{cv\mathbf{k}'}A^*_{cv\mathbf{k}'}\sum_{c'v'\mathbf{k}"}A_{c'v'\mathbf{k}"}\langle 0|c^\dagger_{v\mathbf{k}'}c_{c\mathbf{k}'}c^\dagger_{h\mathbf{k}}c_{h\mathbf{k}}c^\dagger_{c'\mathbf{k}"}c_{v'\mathbf{k}"}|0\rangle \\
&+\sum_{\mathbf{q}\nu}\sum_{ss'}K(E^{s\mathbf{q}}-E^A+\hbar\omega_{\mathbf{q}\nu})K(E^{s'\mathbf{q}}-E^A+\hbar\omega_{\mathbf{q}\nu})G^*_{sA\nu}(0,\mathbf{q})G_{s'A}(0,\mathbf{q}) \\
&\times\sum_{cv\mathbf{k}'}A^{s*}_{c\mathbf{k}'+\mathbf{q}v\mathbf{k}'}\sum_{c'v'\mathbf{k}"}A^{s'}_{c'\mathbf{k}"+\mathbf{q}v'\mathbf{k}"}\langle 0|c^\dagger_{v\mathbf{k}'}c_{c\mathbf{k}'+\mathbf{q}}c^\dagger_{h\mathbf{k}}c_{h\mathbf{k}}c^\dagger_{c'\mathbf{k}"+\mathbf{q}}c_{v'\mathbf{k}"}|0\rangle \\
&= 1-\sum_c|A_{ch\mathbf{k}}|^2 \\
&+\sum_{\mathbf{q}\nu}\sum_{ss'}K(E^{s\mathbf{q}}-E^A+\hbar\omega_{\mathbf{q}\nu})K(E^{s'\mathbf{q}}-E^A+\hbar\omega_{\mathbf{q}\nu})G^*_{sA\nu}(0,\mathbf{q})G_{s'A}(0,\mathbf{q})\sum_{cv\mathbf{k}'}A^{s*}_{c\mathbf{k}'+\mathbf{q}v\mathbf{k}'}A^{s'}_{c\mathbf{k}'+\mathbf{q}v\mathbf{k}'} \\
&-\sum_{\mathbf{q}\nu}\sum_{ss'}K(E^{s\mathbf{q}}-E^A+\hbar\omega_{\mathbf{q}\nu})K(E^{s'\mathbf{q}}-E^A+\hbar\omega_{\mathbf{q}\nu})G^*_{sA\nu}(0,\mathbf{q})G_{s'A}(0,\mathbf{q})\sum_{c}A^{s*}_{c\mathbf{k}+\mathbf{q}h\mathbf{k}}A^{s'}_{c\mathbf{k}+\mathbf{q}h\mathbf{k}}.
\end{aligned}$$

In the first line we discard terms with mismatched phonon numbers. In the last line we have used,

$$\langle 0|c^\dagger_{v\mathbf{k}'}c_{c\mathbf{k}'+\mathbf{q}}c^\dagger_{h\mathbf{k}}c_{h\mathbf{k}}c^\dagger_{c'\mathbf{k}"+\mathbf{q}}c_{v'\mathbf{k}"}|0\rangle = \delta_{cc'}\delta_{\mathbf{k}'\mathbf{k}"}\delta_{vv'}-\delta_{cc'}\delta_{\mathbf{k}'\mathbf{k}"}\delta_{vh}\delta_{\mathbf{k}'\mathbf{k}}\delta_{v'h}.$$

In the long time limit only terms with energy argument matched is nonzero so we have

$$\left\langle \phi \left| c^{\dagger}_{h\mathbf{k}} c_{h\mathbf{k}} \right| \phi \right\rangle = 1 - \sum_{c} |A_{c\mathbf{k}h\mathbf{k}}|^2 + 2\pi t \sum_{\mathbf{q}\nu} \sum_{E^s = E^{s'}} \delta\left(E^{s\mathbf{q}} - E^A + \hbar\omega_{\mathbf{q}\nu}\right) G^*_{s'A}(0,\mathbf{q}) G_{sA\nu}(0,\mathbf{q}) \sum_{cv\mathbf{k}'} A^{s'*}_{c\mathbf{k}'+\mathbf{q}v\mathbf{k}'} A^{s}_{c\mathbf{k}'+\mathbf{q}v\mathbf{k}'}$$
$$- 2\pi t \sum_{\mathbf{q}\nu} \sum_{E^s = E^{s'}} \delta\left(E^{s\mathbf{q}} - E^A + \hbar\omega_{\mathbf{q}\nu}\right) G^*_{s'A}(0,\mathbf{q}) G_{sA\nu}(0,\mathbf{q}) \sum_{c} A^{s'*}_{c\mathbf{k}+\mathbf{q}h\mathbf{k}} A^{s}_{c\mathbf{k}+\mathbf{q}h\mathbf{k}}.$$

Finally, we obtain the valence electron population change rate,

$$\begin{aligned}
\frac{dP_{h\mathbf{k}}}{dt} =& \frac{d\left\langle \phi \left| c^{\dagger}_{h\mathbf{k}} c_{h\mathbf{k}} \right| \phi \right\rangle}{dt} \\
=& 2\pi \sum_{\mathbf{q}\nu} \sum_{E^s = E^{s'}} \delta\left(E^{s\mathbf{q}} - E^A + \hbar\omega_{\mathbf{q}\nu}\right) G^*_{s'A}(0,\mathbf{q}) G_{sA\nu}(0,\mathbf{q}) \sum_{cv\mathbf{k}'} A^{s'*}_{c\mathbf{k}'+\mathbf{q}v\mathbf{k}'} A^{s}_{c\mathbf{k}'+\mathbf{q}v\mathbf{k}'} \\
&- 2\pi \sum_{\mathbf{q}\nu} \sum_{E^s = E^{s'}} \delta\left(E^{s\mathbf{q}} - E^A + \hbar\omega_{\mathbf{q}\nu}\right) G^*_{s'A}(0,\mathbf{q}) G_{sA\nu}(0,\mathbf{q}) \sum_{c} A^{s'*}_{c\mathbf{k}+\mathbf{q}h\mathbf{k}} A^{s}_{c\mathbf{k}+\mathbf{q}h\mathbf{k}}
\end{aligned}$$

In the case with nondegenerate exciton states, we obtain

$$\frac{dP_{h\mathbf{k}}}{dt} = 2\pi \sum_{s\mathbf{q}\nu} \delta\left(E^{s\mathbf{q}} - E^A + \hbar\omega_{\mathbf{q}\nu}\right) |G_{sA\nu}(0,\mathbf{q})|^2 \left(1 - \sum_{c} \left|A^{s}_{c\mathbf{k}+\mathbf{q}h\mathbf{k}}\right|^2\right),$$

which has a physical meaning that once an exciton is scattered to state s with a probability proportional to $|G_{sA\nu}|^2$ it will decrease the valence electron population with a weight corresponding to the module square of the exciton envelope function.

The electron population change rate can be derived similarly. We have

$$\begin{aligned}
c_{c\mathbf{k}} |\phi\rangle =& e^{-iE^A t} c_{c\mathbf{k}} \sum_{cv\mathbf{k}'} A_{cv\mathbf{k}'} c^{\dagger}_{c\mathbf{k}'} c_{v\mathbf{k}'} |0\rangle \\
&- i \sum_{s\mathbf{q}\nu} e^{-iE^A t} K(E^{s\mathbf{q}} - E^A + \hbar\omega_{\mathbf{q}\nu}) G_{sA\nu}(0,\mathbf{q}) c_{c\mathbf{k}} \sum_{cv\mathbf{k}'} A^{s}_{c\mathbf{k}'+\mathbf{q}v\mathbf{k}'} c^{\dagger}_{c\mathbf{k}'+\mathbf{q}} c_{v\mathbf{k}'} |0, 1_{\mathbf{q}\nu}\rangle \\
=& e^{-iE^A t} \sum_{v} A_{c\mathbf{k}v\mathbf{k}} c_{v\mathbf{k}} |0\rangle - i \sum_{sv\mathbf{q}\nu} e^{-iE^A t} K(E^{s\mathbf{q}} - E^A + \hbar\omega_{\mathbf{q}\nu}) G_{sA\nu}(0,\mathbf{q}) A^{s}_{c\mathbf{k}v\mathbf{k}-\mathbf{q}} c_{v\mathbf{k}-\mathbf{q}} |0, 1_{\mathbf{q}\nu}\rangle.
\end{aligned}$$

and the expectation value is

$$\begin{aligned}
\left\langle \phi \left| c^{\dagger}_{c\mathbf{k}} c_{c\mathbf{k}} \right| \phi \right\rangle =& \sum_{v} |A_{c\mathbf{k}v\mathbf{k}}|^2 \\
&+ \sum_{sv\mathbf{q}s'} K(E^{s'\mathbf{q}} - E^A + \hbar\omega_{\mathbf{q}\nu})^* K(E^{s\mathbf{q}} - E^A + \hbar\omega_{\mathbf{q}\nu}) G^*_{s'A\nu}(0,\mathbf{q}) G_{sA\nu}(0,\mathbf{q}) A^{s'*}_{c\mathbf{k}v\mathbf{k}-\mathbf{q}} A^{s}_{c\mathbf{k}v\mathbf{k}-\mathbf{q}}.
\end{aligned}$$

The population change rate is

$$\begin{aligned}
\frac{dP_{c\mathbf{k}}}{dt} =& \frac{d\left\langle \phi \left| c^{\dagger}_{c\mathbf{k}} c_{c\mathbf{k}} \right| \phi \right\rangle}{dt} \\
=& 2\pi \sum_{v\mathbf{q}\nu} \sum_{E^s = E^{s'}} \delta\left(E^{s\mathbf{q}} - E^A + \hbar\omega_{\mathbf{q}\nu}\right) G^*_{s'A\nu}(0,\mathbf{q}) G_{sA\nu}(0,\mathbf{q}) A^{s'*}_{c\mathbf{k}v\mathbf{k}-\mathbf{q}} A^{s}_{c\mathbf{k}v\mathbf{k}-\mathbf{q}}
\end{aligned}$$

Electron-phonon coupling matrix elements

In Fig. S7 we plot a few electron-phonon coupling matrix elements summed over phonon mdoes, $\sum_\nu |g_{nm\nu}(\mathbf{K},\mathbf{q})|$ which describes the coupling from band n at $\mathbf{K}$ point to band m at $\mathbf{K}+\mathbf{q}$. For bands around $\mathbf{K}$ point due to the well defined spin characters we can see spin selection rule dictates electron-phonon coupling matrix elements.

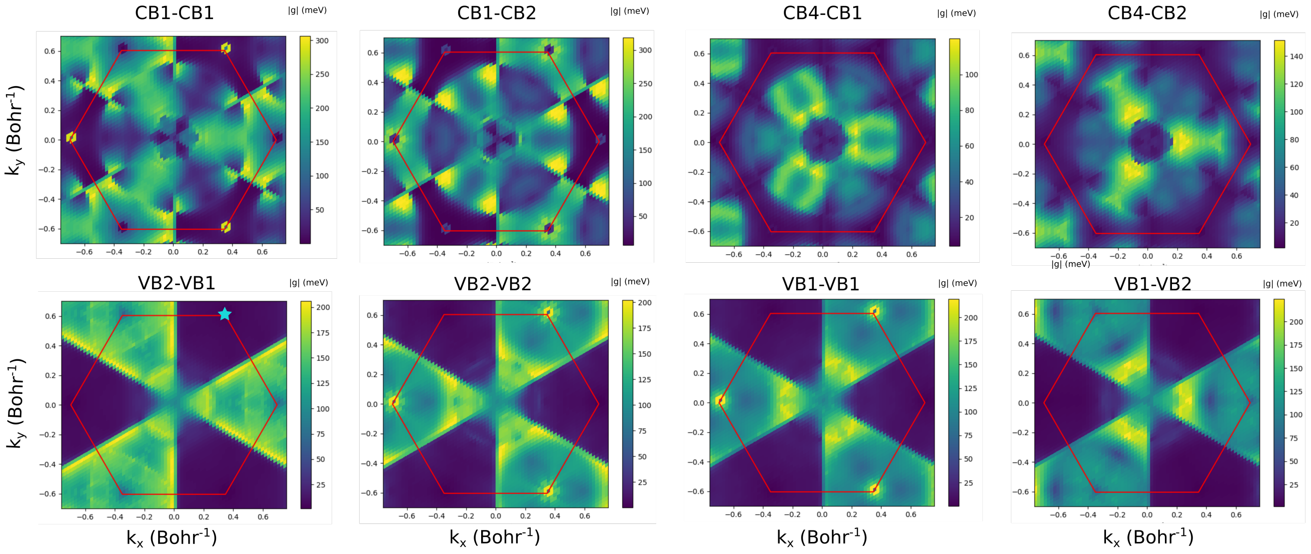

FIG. S7. Phonon-mode summed electron-phonon coupling matrix elements $\sum_\nu |g_{nm\nu}(\mathbf{K},\mathbf{q})|$ for various pair of bands starting from $\mathbf{K}$ (Blue star) to other $\mathbf{k}$-point in the BZ. The band index is counted from the Fermi level.

* yanghao@gate.sinica.edu.tw
† diana.qiu@yale.edu
‡ jornada@stanford.edu

[1] Paolo Giannozzi, Stefano Baroni, Nicola Bonini, Matteo Calandra, Roberto Car, Carlo Cavazzoni, Davide Ceresoli, Guido L Chiarotti, Matteo Cococcioni, Ismaila Dabo, Andrea Dal Corso, Stefano de Gironcoli, Stefano Fabris, Guido Fratesi, Ralph Gebauer, Uwe Gerstmann, Christos Gougoussis, Anton Kokalj, Michele Lazzeri, Layla Martin-Samos, Nicola Marzari, Francesco Mauri, Riccardo Mazzarello, Stefano Paolini, Alfredo Pasquarello, Lorenzo Paulatto, Carlo Sbraccia, Sandro Scandolo, Gabriele Sclauzero, Ari P Seitsonen, Alexander Smogunov, Paolo Umari, and Renata M Wentzcovitch, "Quantum espresso: a modular and open-source software project for quantum simulations of materials," Journal of Physics: Condensed Matter **21**, 395502 (2009).

[2] J. P. Perdew and Alex Zunger, "Self-interaction correction to density-functional approximations for many-electron systems," Phys. Rev. B **23**, 5048–5079 (1981).

[3] D. R. Hamann, "Optimized norm-conserving vanderbilt pseudopotentials," Phys. Rev. B **88**, 085117 (2013).

[4] M.J. van Setten, M. Giantomassi, E. Bousquet, M.J. Verstraete, D.R. Hamann, X. Gonze, and G.-M. Rignanese, "The pseudodojo: Training and grading a 85 element optimized norm-conserving pseudopotential table," Computer Physics Communications **226**, 39–54 (2018).

[5] Mark S. Hybertsen and Steven G. Louie, "Electron correlation in semiconductors and insulators: Band gaps and quasiparticle energies," Phys. Rev. B **34**, 5390–5413 (1986).

[6] Michael Rohlfing and Steven G. Louie, "Electron-hole excitations and optical spectra from first principles," Phys. Rev. B **62**, 4927–4944 (2000).

[7] Jack Deslippe, Georgy Samsonidze, David A. Strubbe, Manish Jain, Marvin L. Cohen, and Steven G. Louie, "Berkeleygw: A massively parallel computer package for the calculation of the quasiparticle and optical properties of materials and nanostructures," Computer Physics Communications **183**, 1269–1289 (2012).

[8] Felipe H. da Jornada, Diana Y. Qiu, and Steven G. Louie, "Nonuniform sampling schemes of the brillouin zone for many-electron perturbation-theory calculations in reduced dimensionality," Phys. Rev. B **95**, 035109 (2017).

[9] Changpeng Lin, Samuel Poncé, and Nicola Marzari, "General invariance and equilibrium conditions for lattice dynamics in 1d, 2d, and 3d materials," npj Computational Materials **8**, 236 (2022).

[10] Feliciano Giustino, Marvin L. Cohen, and Steven G. Louie, "Electron-phonon interaction using wannier functions," Phys. Rev. B **76**, 165108 (2007).

[11] S. Poncé, E.R. Margine, C. Verdi, and F. Giustino, "Epw: Electron–phonon coupling, transport and superconducting properties using maximally localized wannier functions," Computer Physics Communications **209**, 116–133 (2016).

[12] Yutaka Toyozawa, "Theory of Line-Shapes of the Exciton Absorption Bands," Progress of Theoretical Physics **20**, 53–81 (1958), https://academic.oup.com/ptp/article-pdf/20/1/53/5457877/20-1-53.pdf.

[13] Gabriel Antonius and Steven G. Louie, "Theory of exciton-phonon coupling," Phys. Rev. B **105**, 085111 (2022).

[14] Hsiao-Yi Chen, Davide Sangalli, and Marco Bernardi, "Exciton-phonon interaction and relaxation times from first principles," Phys. Rev. Lett. **125**, 107401 (2020).

[15] Yang-hao Chan, Jonah B. Haber, Mit H. Naik, Jeffrey B. Neaton, Diana Y. Qiu, Felipe H. da Jornada, and Steven G. Louie, "Exciton lifetime and optical line width profile via exciton-phonon interactions: Theory and first-principles calculations for monolayer mos2," Nano Lett. (2023), 10.1021/acs.nanolett.3c00732.

[16] Xiaoping Hong, Jonghwan Kim, Su-Fei Shi, Yu Zhang, Chenhao Jin, Yinghui Sun, Sefaattin Tongay, Junqiao Wu, Yanfeng Zhang, and Feng Wang, "Ultrafast charge transfer in atomically thin mos2/ws2 heterostructures," Nature Nanotechnology **9**, 682–686 (2014).

[17] Engin Torun, Henrique P. C. Miranda, Alejandro Molina-Sánchez, and Ludger Wirtz, "Interlayer and intralayer excitons in mos_2/ws_2 and $mose_2/wse_2$ heterobilayers," Phys. Rev. B **97**, 245427 (2018).

[18] Andrea Marini, "Ab initio finite-temperature excitons," Phys. Rev. Lett. **101**, 106405 (2008).

[19] Alejandro Molina-Sánchez, Maurizia Palummo, Andrea Marini, and Ludger Wirtz, "Temperature-dependent excitonic effects in the optical properties of single-layer mos_2," Phys. Rev. B **93**, 155435 (2016).

[20] Albert F. Rigosi, Heather M. Hill, Yilei Li, Alexey Chernikov, and Tony F. Heinz, "Probing interlayer interactions in transition metal dichalcogenide heterostructures by optical spectroscopy: Mos2/ws2 and mose2/wse2," Nano Lett. **15**, 5033–5038 (2015).

[21] D.W. Snoke, "The quantum boltzmann equation in semiconductor physics," Annalen der Physik **523**, 87–100 (2011), https://onlinelibrary.wiley.com/doi/pdf/10.1002/andp.201000102.

[22] Hsiao-Yi Chen, Davide Sangalli, and Marco Bernardi, "First-principles ultrafast exciton dynamics and time-domain spectroscopies: Dark-exciton mediated valley depolarization in monolayer wse_2," Phys. Rev. Res. **4**, 043203 (2022).

[23] Alejandro Molina-Sánchez, Davide Sangalli, Ludger Wirtz, and Andrea Marini, "Ab initio calculations of ultrashort carrier dynamics in two-dimensional materials: Valley depolarization in single-layer wse2," Nano Lett. **17**, 4549–4555 (2017).

[24] Gianluca Stefanucci and Robert van Leeuwen, *Nonequilibrium Many-Body Theory of Quantum Systems: A Modern Introduction* (Cambridge University Press, 2013).
